# Need for a gender-sensitive human security framework: results of a quantitative study of human security and sexual violence in Djohong District, Cameroon

**DOI:** 10.1186/1752-1505-8-6

**Published:** 2014-05-07

**Authors:** Parveen Kaur Parmar, Pooja Agrawal, Ravi Goyal, Jennifer Scott, P Gregg Greenough

**Affiliations:** 1 Harvard Humanitarian Initiative, Harvard University, Cambridge, MA, USA; 2 Division of Emergency Medicine, Harvard Medical School, Brigham & Women’s Hospital, Boston, MA, USA; 3 Global Health and Population, Harvard School of Public Health, Boston, MA, USA; 4 Division of Emergency Medicine, Yale School of Medicine & Yale New Haven Hospital, New Haven, CT, USA; 5 Department of Biostatistics, Harvard School of Public Health, Boston, MA, USA; 6 Department of Obstetrics and Gynecology, Harvard Medical School, Beth Israel Deaconess Medical Center, Boston, MA, USA; 7 Division of Women' Health, Harvard Medical School, Brigham and Women's Hospital, Boston, MA, USA

**Keywords:** Sexual violence, Human security, Women’s health, Cameroon, Central African Republic, Refugee

## Abstract

**Background:**

Human security shifts traditional concepts of security from interstate conflict and the absence of war to the security of the individual. Broad definitions of human security include livelihoods and food security, health, psychosocial well-being, enjoyment of civil and political rights and freedom from oppression, and personal safety, in addition to absence of conflict.

**Methods:**

In March 2010, we undertook a population-based health and livelihood study of female refugees from conflict-affected Central African Republic living in Djohong District, Cameroon and their female counterparts within the Cameroonian host community. Embedded within the survey instrument were indicators of human security derived from the Leaning-Arie model that defined three domains of psychosocial stability suggesting individuals and communities are most stable when their core attachments to home, community and the future are intact.

**Results:**

While the female refugee human security outcomes describe a population successfully assimilated and thriving in their new environments based on these three domains, the ability of human security indicators to predict the presence or absence of lifetime and six-month sexual violence was inadequate. Using receiver operating characteristic (ROC) analysis, the study demonstrates that common human security indicators do not uncover either lifetime or recent prevalence of sexual violence.

**Conclusions:**

These data suggest that current gender-blind approaches of describing human security are missing serious threats to the safety of one half of the population and that efforts to develop robust human security indicators should include those that specifically measure violence against women.

## Background

### Human security: background

Human security shifts the traditional concept of security from interstate conflict and the absence of war to the security of the individual [1]. In doing so, it recognizes that ‘the basic rights of people, not merely the absence of military conflict between states, are fundamental to world stability [2]’. Despite that clarity, concepts of human security vary greatly. Some authors advocate for a broad definition, including threats to livelihoods and food security, health, psychosocial well-being, enjoyment of civil and political rights and freedom from oppression, and personal safety, in addition to absence of conflict. While inclusive, this broad definition can be unwieldy and difficult to operationalize [3,4]. King and Murray emphasize freedom from violence, advocating for a narrower definition that includes those factors which are ‘important enough for human beings to fight over or to put their lives or property at great risk’, and identify five key indicators: poverty, health, education, political freedom, and democracy [5]. However, as pointed out by Paris in 2001, narrower definitions risk excluding important threats to personal security that do not relate to these five indicators [3]. Hastings developed a Human Security Index that attempts to move beyond United Nations Development Programme’s (UNDP) Human Development Index to include various national measures of stability and inequity in society (including gender, environment, health, a ‘social fabric index’, and measures of corruption). His approach, however, does not include a measure of violence against women, a measure he suggests is a ‘women’s health issue’ as opposed to a human security issue [6]. Authors have posited that a ‘gender-blind’ approach to human security will similarly miss important threats to individual and community security [7-9].

Multiple authors have explored the role of inequality and oppression in inter- and intrastate violence. In general, studies have found that greater social disparities between segments of society are associated with greater levels of conflict [8,10,11]. The role of gender inequity in conflict has also been previously explored. Melander found that states with higher rates of gender inequity, as measured by female participation in parliament and female to male higher education attainment ratio, have higher rates of intrastate conflict [12]. Similarly, Caprioli found that states with higher levels of gender inequity had higher rates in intrastate violence between 1960 and 2001 [8].

Assessing levels of human security remains challenging. Given the links described between human security indicators and a greater propensity to intra- and interstate conflict, measurement of human security could be used both to herald potential deterioration within a conflict and to monitor progress of post-conflict state conditions. Leaning and Arie in 2001 [10] developed a model designed for this purpose. Based on experiential knowledge and extensive qualitative review of human security as understood in African populations that have been insecure, the Leaning-Arie model argues that a population requires a ‘core bundle of basic resources: material psychological and social’ to ensure a minimum level of survival. ‘If minimum material inputs can be guaranteed and if efforts can be made to shore up basic social coping capacities, societies will be more stable and less prone to fragmentation, violence and atrocity’ [10]. This model moves beyond measurement of basic material supports (food, water, shelter, income) to incorporate critical psychosocial components associated with individual security: namely, ‘a sense of home and safety, constructive family and social supports, and an acceptance of the past and positive grasp of the future [10]’. These three domains of home, community, and positive sense of future reflect the ways humans relate to themselves, each other, and the world: ‘[humans] are anchored and seek safety in a sense of home; they find meaning, identity, and functional support in their relationships with family and community; and they build their lives through time, depending upon their sense of trust in the future and their sense of freedom from the past [10]’. The main strength of this model is its ability to detect threats to human security at a more granular level that may be used to predict community level violence—a key difference from other approaches that focus on aggregate national indicators. This model, however, does not explicitly incorporate gender sensitive measures.

### Cameroon

The following study explores human security and gender using quantitative measures in Djohong District, Cameroon, a rural region to where members of the Mbororo tribe from the Central African Republic (CAR) have fled in response to a decade of targeted killings, kidnappings, forced taxation and extortion, beatings, and the burning and looting of their homes and villages [13,14]. These human rights violations have occurred at the hands of government troops, anti-government rebel groups, and opportunistic gangs of bandits known locally as *coupeurs de route*[15]. At the time of this study, an estimated 80,000 had fled across CAR’s western border into Cameroon [13], 21,000 of whom, as of 2010, lived as refugees in the sparsely populated and mainly agricultural Djohong. During their flight, the *coupeurs de route* targeted the Mbororo, locally perceived as wealthy pastoralists, stealing their livestock or kidnapping their family members and forcing them to pay large ransoms. Arriving in Djohong with little or no assets, the Mbororo refugees had become considerably dependent on the international aid community and Cameroonian host population. In early 2007, the United Nations Office for the Coordination of Humanitarian Affairs (UNOCHA) cited a high burden of sexual violence in the internally displaced and refugee population, but detailed statistics were lacking [16].

We examined human security indicators based on the Leaning-Arie model and performed a population-based study for measures of sexual and gender based violence. From this, we used the model to determine whether a human security framework that does not specifically incorporate measures related to gender would capture insecurity faced by women affected by displacement and recent conflict, and to assess whether or not gender-specific measures are needed when measuring human security.

## Methods

In March 2010, we undertook a population-based study conducted in Djohong District, Cameroon to better ascertain women’s health needs within the refugee and Cameroonian host communities [17]. Embedded within the survey instrument were indicators of human security derived from the Leaning-Arie model, assessing three domains of psychosocial stability that suggest individuals and communities are most stable when their core attachments to home, community and the future are intact. The Leaning-Arie model was selected as it was designed to be used for community-level, ongoing measurement of human security that could be used to assess a community’s tendency toward instability and conflict over time [10]. The domain of sense of attachment to home includes a ‘sustainable sense of home and safety providing identity, recognition, and freedom from fear’ measured as 1) number of years in current location; 2) proportion of the population who own land; and 3) self-reported sense of safety. Sense of attachment to community, the second domain in the model, elaborates the individual’s relationship to their community as ‘a network of constructive social or familial support, providing identity, recognition, participation and autonomy’ [10], such that sudden changes in economic or political circumstances, or perceived inequalities relative to others that appear not to be improving can potentially precipitate violent behavior. Indicators used to measure attachment to community include 1) measures of dynamic inequality between members of different groups, including income and assets; and 2) subjective sense of ties to and inclusion in one’s community or village. The third domain is a positive grasp of the future which includes ‘the confidence that one’s own capacities, and the external social and political structures that confer meaning and stability, will persist for some indefinite period into the future’ [10]. This can be measured by an individual’s plans to remain in the current village, raise children in the current village, and plans to plant for future harvests. The text below outlines these three domains and provides examples of how each was specifically studied.

### **Human security framework: Leaning Arie model**

Attachment to Home:

• Number of years living in this village

• Does any extended family live in this village?

• Do you own land?

• Do you feel safe in this village?

Attachment to Community:

• Is your home made from mud brick or grass/reeds/sticks/tarp?

• How much land do you own? How much livestock do you own?

• Do you feel attached to the community in this village?

• If you needed medical care, would someone take you to the hospital?

Positive outlook for the Future:

• Do you want to grow old and die in this village?

• Will your children be working in this village in 10 years?

• Will you be using your land in 2 years?

• Do you plan to stay in this village?

### Sampling strategy

Female refugee and host population participants were selected using a two-stage household cluster design. The first stage included a random selection of 40 clusters (villages) weighted by refugee and host population estimates from the UN High Commissioner for Refugees (UNHCR) registration lists of January 2010 and from Djohong District government officials, respectively. The latter figures were 2010 estimates of the Cameroonian population using 2006 census figures adjusted for annual population growth. Since the human security dataset came from a study designed to also characterize gender-based violence (GBV) in the region, the estimated prevalence of GBV was used to determine sample size. Based on an estimated prevalence of 20-30% from previous GBV studies in Africa [18], a desired precision of 0.05, and a design effect of 2.0, we estimated a sample size between 500 and 650, assuming a non-response rate of 5%. Since observations within a cluster may be more alike than observations across clusters, particularly with shared perceptions of human security, we took this intra-cluster correlation into account in the sample size calculation. This design effect, defined as the ratio of the variance taking into account the cluster sample design and variance of a simple random sample design with the same number of observations, was conservatively estimated at 2.0 based both on previous sexual violence studies [19,20]. To minimize the possibility of increased intra-cluster correlation and homogeneity on the outcome of sexual violence prevalence and to limit design effect, we sampled more clusters in the first stage and fewer households in the second stage [21]; the second stage included 15 randomly selected women per cluster for a final sample size of 600 respondents.

In stage one, clusters were defined as villages. To account for the modest variation in village populations across the district, probability proportional to size (PPS) sampling was used to randomly select clusters. As the refugee population does not live in segregated areas but is interspersed among the host Cameroonian villages [13], we reasoned that random sampling in each village would result in a sample demographic similar to the population demographic. All villages in Djohong District were considered for possible selection. The World Health Organization’s (WHO) Extended Program on Immunization (EPI) method [22] was used in the second stage to randomly select the 15 respondent households in each village. A household was defined as a group of individuals living under the same roof and eating meals from the same pot. Village chiefs assisted the research team to identify the geographic center of each village selected. From that point, the team randomly selected a direction by spinning a pen on a flat surface and then randomly selected a number of houses to pass to reach the first sampled household. Each subsequent household whose door was nearest to the door of the previous household was surveyed until all 15 surveys within the cluster were completed. To minimize non-response, a pre-visit announcement was sent to each village cluster to request the presence of all adult women in the village for the day of sampling. Three attempts were made to contact selected households where respondents were initially unavailable. When, as a result of PPS sampling, larger villages contained more than one cluster, clusters were geographically distributed according to the location of population centers.

### Survey

At each village cluster, the study was announced as a women’s health study for purposes of safety and confidentiality. The team queried the adult female (≥18 years of age) head of household on household demographics, human security, household economy and assets, level of education, food security, water, fuel, shelter, access to health care, self-reported mental health, self-reported reproductive health and sexual violence experienced by the respondent only. In polygamous households, the senior wife was interviewed, a decision based on cultural custom.

For the purposes of this study, sexual violence was defined as any physically or verbally forced sexual act, including molestation, forced undressing, forced or unwelcome touching of a sexual nature, forced intercourse, forced insertion of an object into any body cavity or any other non-consensual sexual act, whether completed or not.

Perpetrators were defined as the person or persons responsible for the forced act. Refugees were defined according to the UNHCR definition: ‘owing to a well-founded fear of being persecuted for reasons of race, religion, nationality, membership of a particular social group or political opinion, is outside the country of his nationality, and is unable to, or owing to such fear, is unwilling to avail himself of the protection of that country [23]’. Given considerations of confidentiality, no identification or proof was used to verify refugee status.

The survey instrument, much of which had been field tested the previous year for a baseline prevalence study on sexual violence, was translated and back-translated by bilingual French-English speakers in Cameroon, then tested and colloquially adjusted by data collectors who were local health professionals (with the Cameroonian Ministry of Health at Djohong Hospital) as well as local community health workers on staff with an international health non-governmental organization (NGO). Training included detailed explorations of each question with fine-tuning of terms and translation in French and in the local language, Fulfulde. Data collectors were then trained on the sampling methodology, including the EPI method, using simulated models of villages with varying configurations. Following training on the survey instrument, interviews were conducted in Fulfulde in a setting that ensured privacy and confidentiality.

### Human subjects protection

This study was conducted in accordance with the WHO ethical and safety recommendations for researching, documenting and monitoring sexual violence in emergencies [24]. Due to high rates of illiteracy, the team obtained oral consent prior to administration of the survey and specifically informed the respondents that the survey would ask about sexual violence as a women’s health and livelihood concern. Prior to survey questions on sexual violence, a second verbal consent was obtained to allow the participant an opportunity to refuse to answer questions in this section. Respondents were assured that their names would not be recorded, that there would be no penalties or benefits for refusing or agreeing to participate, and were offered access to counseling and medical services through the international NGO and local providers as needed. The study was reviewed and approved by the Office of Human Research Administration at the Harvard School of Public Health. All male data collectors were required to have previous experience in the care of female survivors of sexual violence, either as clinicians or as sexual violence counselors. While WHO guidelines recommend female surveyors and translators whenever possible, the lack of female staff and the availability of experienced sexual violence male counselors in the region necessitated their inclusion as surveyors. The survey instrument did not identify respondents.

### Analysis

The lead field investigators checked the data for errors, then coded and entered it daily into a password protected Excel spreadsheet. Non-identifiable hard copies were stored in a locked facility on the NGO compound in Djohong District. At the end of the survey, the lead investigators securely transported hard copies to a locked storage facility at the Harvard Humanitarian Initiative where they remained accessible only to the lead investigators. STATA 11 (StataCorp, College Station, TX) was used for the analysis of the imported spreadsheet. Cluster sampling design was accounted for in the analysis; a generalized Hansen-Hurwitz estimator for a two-stage cluster design was used to estimate the means and percentages and confidence intervals were constructed by calculating the standard error of the generalized Hansen-Hurwitz estimator. Receiver operating curve (ROC) analysis was used to evaluate the predictive value of human security indicators for both lifetime and six-month sexual violence. Three predictive models were analyzed for their ability to predict sexual violence within the refugee and host populations: 1) age and ethnicity; 2) age, ethnicity, time in village, and all human security indicators; and 3) age, ethnicity, time in village and community human security indicators only.

## Results

### Demographics

Demographic results for the population in Eastern Cameroon are given in Table 1. Of the 600 randomly selected households, three declined to participate, for an overall response rate of 99.5%. Due to prior notification in each village of the upcoming survey, all female heads of household were present on the days of data collection; all targeted respondents were reached within three visits. Surveyed adult female heads of households included 397 Cameroonians, 191 refugees, eight who classified themselves as ‘other’, and one who did not respond. Because the study involves comparison of refugee and Cameroonian populations, the nine who either classified themselves as ‘other’ or did not identify in either group were removed from the analysis. The final sample resulted in 32.5% refugees (95% CI 23.3 - 41.7) and 67.5% Cameroonians (95% CI 58.3 - 76.7), which closely matched the population distribution based on census data from the local government and the UNHCR registration rolls (31% refugee, 69% host population). Respondents ranged from 17 to 99 years of age; mean age of refugee respondents was 35.1 years (95% CI 32.8 - 37.4), while the mean age of Cameroonian respondents was 34.5 years (95% CI 32.7 - 36.2). The mean size of refugee households was 6.6 persons (95% CI 6.0 - 7.2, range 1–17) and the mean size of Cameroonian households was 5.9 persons (95% CI 5.6 - 6.2, range 1–17).

**Table 1:** Respondent characteristics

**Characteristic**	**Refugees n = 191**	**Cameroonian n = 397**
Proportion of respondents*	32.5 (23.3 – 41.7)	67.5 (58.3 – 76.7)
Mean age	35.1 (32.8 – 37.4)	34.5 (32.7 – 36.2)
Mean household size	6.6 (6.0 – 7.2)	5.9 (5.6 – 6.2)

Data presented as mean % and weighted % (95% CI).

*Total of 588 respondents.

### Human security

#### ***Sense of attachment to home***

Outcomes of respondent’s attachment to home indicators are summarized in Table 2. As expected, refugee respondents had spent significantly less time in their current village than host population respondents: for refugees, a mean of 3.9 years (95% CI 3.2 - 4.5) as compared to 17.9 years (95% CI 15.7 - 20.1) for host population, or Cameroonian women. Similarly, a slightly larger proportion of Cameroonian women had extended family in the current village (44.7%, 95% CI 36.8 – 52.7 of refugees vs. 59.8% 95% CI 52.8 – 66.9 of Cameroonians), and a significantly larger proportion of Cameroonian households owned land (refugees 68.3%, 95% CI 56.4 – 80.1 vs. Cameroonians 87.6%, 95% CI 81.8 – 93.3). Despite these disparities, greater than 95% of both refugee and host community women reported feeling safe in their current village, and these proportions did not differ between populations (98.4%, 95% CI 96.8 – 100.0 of refugees vs. 95.7%, 95% CI 93.4 - 98.0 of Cameroonians).

**Table 2:** Human security outcomes: sense of attachment to home

**Characteristic**	**Refugee (n = 190)**	**Refugee % (95% CI)**	**Cameroonian (n = 396)**	**Cameroonian % (95% CI)**
Years in village	190	3.9 (3.2 – 4.5)	396	17.9 (15.7 – 20.1)
Does your extended family live in this village?	85	44.7 (36.8 – 52.7)	237	59.8 (52.8 – 66.9)
Do you own land?	129*	68.3 (56.4 – 80.1)	345^	87.6 (81.8 – 93.3)
I feel safe in this village	188±	98.4 (96.8 – 100)	379	95.7 (93.4 – 98.0)

Data presented as mean % and weighted % (95% CI) of those who stated ‘yes’, with exception of first row reporting years, as noted.

*of 189 refugees.

^of 394 Cameroonians.

±of 191 refugees.

#### ***Sense of attachment to community***

Though the proportions varied between refugee and Cameroonian host population, the majority of both belonged to the same four ethnic tribes and shared common religions (Table 3). However, of the refugee respondents, 83.3% (95% CI 74.2 - 89.6) were Muslim and 16.8% (95% CI 10.4 - 25.8) were Christian, while 47.9% of the host population was Muslim (95% CI 36.2 - 59.7) and 52.1% (95% CI 40.3 - 63.8) was Christian.

**Table 3:** Human security outcomes: sense of attachment to community, ethnicity

**Characteristic**	**Refugee (n = 191)**	**Refugee % (95% CI)**	**Cameroonian (n = 397)**	**Cameroonian % (95% CI)**
**Ethnicity**				
*Mbororo*	106	55.5 (43.8 – 66.6)	39	9.8 (6.3 – 15.1)
*Gbaya*	27	14.1 (9.3 – 21.0)	214	53.9 (40.2 – 67.1)
*Fulbe*	20	10.5 (5.5 – 19.0)	83	20.9 (13.3 – 31.4)
*Poulo*	21	11.0 (5.5 – 20.9)	22	5.5 (3.2 – 9.5)
*Pana*	6	3.1 (1.0 – 9.5)	10	2.5 (0.9 – 6.4)
*Other*	11	5.8 (2.8 – 11.6)	29	7.3 (3.6 – 14.2)
**Religion**				
*Muslim*	159	83.3 (74.2 – 89.6)	190	47.9 (36.2 – 59.7)
*Christian*	32	16.8 (10.4 – 25.8)	207	52.1 (40.3 – 63.8)

Household assets included the type of home (‘mud brick’ considered best quality; ‘grass, reeds, sticks, or tarp’ the least quality and most temporary). A high proportion of both refugees and host population lived in more permanent mud brick structures (refugees 95.3%, 95% CI 90.6 – 100.0 vs. Cameroonian 98.0%, 95% CI 96.5 – 99.5, Table 4). Each population reported similar levels of animal assets (numbers of cows and chickens owned), and the average size of land owned among those who owned land (Table 5). Thus, though a higher proportion of Cameroonians owned land, when refugees owned land they owned land in similar amounts, (Table 2). A relatively large proportion of refugees in eastern Cameroon reported land ownership. As shown in Table 5, both refugees and host population reported a mean of 1.7 days without food in the past month, suggesting non-differential access to food.

**Table 4:** Human security outcomes: sense of attachment to community, home

**Characteristic**	**Refugee (n = 191)**	**Refugee % (95% CI)**	**Cameroonian (n = 396)**	**Cameroonian % (95% CI)**
Water comes from a protected well	107	56.0 (43.2 – 68.9)	137	34.6 (22.9 – 46.3)
Home is a made from mud brick	182	95.3 (90.6 – 100)	388	98.0 (96.5 – 99.5)
Home is made of grass, reeds, sticks or tarp	9	4.7 (0 – 9.4)	8	2.0 (0 – 3.5)

Data presented as mean % and weighted % (95% CI).

**Table 5:** Human security outcomes: sense of attachment to community, assets

**Characteristic**	**Refugee n**	**Refugee mean (95% CI)**	**Cameroonian n**	**Cameroonian mean (95% CI)**
Average daily income, CFA	188	842.38 (593.58 – 1091.18)	394	1172.53 (1001.66 – 1343.39)
Average size of land owned, hectares	187	1.54 (0.38 – 2.69)	397	1.26 (0.68 – 1.83)
Cows owned by household	191	6.6 (0.2 – 12.9)	396	6.2 (3.9 – 8.5)
Chickens owned by household	191	2.3 (1.5 - 3.2)	396	2.3 (1.6 – 3.0)
Day without food in the past month	191	1.7 (1.6 – 1.8)	395	1.7 (1.7 – 1.8)

Though confidence intervals overlap, Cameroonian households had a higher average daily income (refugee 842.38 CFA, 95% CI 593.58 – 1091.18 vs Cameroonian 1172.53 CFA, 95% CI 1001.66 – 1343.39). Refugees had access to protected wells in greater proportions (56.0% 95% CI 43.2 – 68.9) than the host population (34.6% 95% CI 22.9 – 46.3) (Table 4).

Although both refugee and host populations reported subjective measures of attachment to their community and forming strong relationships within their community in similarly high proportions (Table 6), when asked concretely whether they could rely on members of their community for assistance, answers differed: though confidence intervals overlapped in many cases. While 30.2% (95% CI 20.5 – 39.8) of refugees believed a non-community family member would help them with money if they needed it, 47.0% (95% CI 39.6 – 54.3) of Cameroonians believed they could reach out to members of their community for financial assistance. When asked if they could rely on someone in the village for help if they were alone, 39.6% (95% CI 29.4 – 49.7) of refugees reported they could while a higher proportion of Cameroonians, 56.2% (95% CI 49.5 – 62.9), believed a community member would come to their aid. When asked specifically if a non-family villager would take them to the hospital if they became sick, a lower proportion of refugees believed they would (40.4%, 95% CI (29.5 – 51.4) compared to the native Cameroonians (59.7%, 95% CI 52.3 – 67.1).

**Table 6:** Human security outcomes: sense of attachment to the community, perceptions in the affirmative

**Characteristic**	Refugee*	Refugee % (95% CI)**	Cameroonian*	Cameroonian % (95% CI)**
If I was alone in this village someone would help	74 (187)	39.6 (29.4 – 49.7)	222 (395)	56.2 (49.5 – 62.9)
If I needed money, someone in this village other than family would help	57 (189)	30.2 (20.5 – 39.8)	185 (394)	47.0 (39.6 – 54.3)
If I needed urgent medical care, non-family villagers would take me to the hospital	76 (188)	40.4 (29.5 – 51.4)	234 (392)	59.7 (52.3 – 67.1)
I feel attached to the community in this village	173 (191)	90.6 (86.1 – 95.1)	359 (395)	90.9 (86.2 – 95.6)
The members of my community form strong relationships and rely on each other for support	134 (190)	70.5 (61.3 – 79.8)	299 (396)	75.5 (68.0 – 83.0)

*Data presented as # of affirmative responses (# total responses).

**Data presented as mean % and weighted % (95% CI).

#### ***Positive grasp of the future***

Though measures of attachment to community differed between refugees and Cameroonians, both had similarly high measures with respect to a positive sense of future. Similar proportions of refugees and Cameroonians indicated that they would like to ‘grow old’ in their current village, that their children would live in their current village in ten years, and that their children would work in their current village in ten years (Table 7). Over 98% of both refugees and Cameroonians believed they would be using their land in two years, and over 90% plan to stay in their current village. These data suggest that the majority of refugees in Eastern Cameroon were not planning to return to CAR at the time of this study.

**Table 7:** Human security outcomes: sense of future

**Characteristic**	Refugee*	Refugee % (95% CI)**	Cameroonian*	Cameroonian % (95% CI)**
I want to grow old and die in this village	159 (191)	83.2 (77.7 – 88.8)	322 (395)	81.5 (76.6 – 86.5)
My children will live in this village in 10 years	158 (190)	83.2 (77.3 – 89.0)	297 (389)	76.3 (70.8 – 81.9)
My children will work in this village in 10 years	141 (190)	74.2 (66.3 – 82.1)	247 (389)	63.5 (55.5 – 71.5 )
I will use my land in 2 years	153 (156)	98.1 (97.2 – 99.8)	325 (330)	98.5 (96.0 – 100)
I plan to stay here	172 (188)	91.5 (86.8 – 96.2)	354 (389)	91.0 (87.8 – 94.2)

*Data presented as # of affirmative responses (# total responses).

**Data presented as mean % and weighted % (95% CI).

### Sexual violence

Female heads of household were asked whether or not they had experienced sexual violence during their lifetimes or during the past six months. A complete report on the prevalence of sexual violence in this population has been previously published [17]; a selection of these data are presented here to highlight the context of human security measures.

Lifetime prevalence of sexual violence among all Djohong district female heads of household was 35.2% (95% CI 28.7 - 42.2), 206 of the 597 respondents (Table 8). Among the female refugee population, 40.8% (95% CI 31.5 - 50.8) reported at least one episode of sexual violence during their lifetimes, and 32.4% (95% CI 25.2 - 40.5) of Cameroonian female heads of household reported at least one episode of sexual violence during their lifetimes (Table 9). The prevalence of sexual violence over the last six months (of those women who reported a sexually violent event) was nearly identical: 39.0% (95% CI 21.7 - 59.6) for refugee women and 38.3% (95% CI 28.4 - 49.2) for Cameroonian women.

**Table 8:** Sexual violence, total population

**Characteristic**	**# with characteristic**	**Total respondents**	Weighted % (95% CI)*
Lifetime prevalence	206**	589	35.2 (28.7 – 42.2)
**Perpetrator**			
Husband	131	205**	64.0 (54.3 – 72.5)
Friend or Member of the Community	41	205	20.0 (14.2 – 27.5)
Soldier/rebel or coupeurs de route***	35	205	17.1 (10.7 – 26.1)
Unknown	6	205	2.9 (1.2 – 7.2)
Estimated population-wide sexual violence by partner/husband	131	588	22.3 (17.1 – 28.5)

*Data presented as mean % and weighted % (95% CI).

**206 women answered ‘yes’ when asked about sexual violence, however one respondent declined to provide any further information. Thus, further analyses are on 205 respondents.

***8 respondents reported both soldiers/rebel and *coupers de route* with regards to episodes of sexual violence by multiple perpetrators. Soldiers/rebels and *coupers de route* were combined based on qualitative data collected by the authors that suggests overlap among these groups in Northwest CAR.

**Table 9:** Sexual violence, broken down by Refugee vs Cameroonian

**Characteristic**	**Refugee n = 77**	Refugee % (95% CI)*	**Cameroonian n = 128**	Cameroonian % (95% CI)*
Lifetime prevalence	78** (191)	40.8 (31.5 – 50.8)	128 (395)	32.4 (25.2 – 40.5)
Sexual violence in the past 6 months	30	39.0 (21.7 – 59.6)	49	38.3 (28.4 – 49.2)
**Perpetrator**				
Husband	40	52.0 (35.4 – 68.1)	91	71.1 (61.0 – 79.5)
Member of my family	0		1	0.8 (0–6)
Friend or Member of the Community	12	15.6 (8.3 – 27.2)	28	21.2 (13.9 – 32.8)
Soldier/rebel or coupeurs de route***	30	39.0 (25.6 – 54.2)	5	3.9 (1.4 – 10.5)
Unknown	3	3.9 (1.3 – 11.5)	3	2.3 (0.7 – 7.3)
Multiple perpetrators	29	37.7 (22.9 – 52.5)	8	6.3 (1.6 – 10.9)

*Data presented as mean % and weighted % (95% CI).

**The above respondent identified as ‘refugee’, thus further analyses are on 77 refugees only.

***8 people said both soldiers/rebel and *coupers de route* in a multiple perp sexual violence. Soldiers/rebels and *coupers de route* were combined based on qualitative data collected by the authors that suggests overlap among these groups in Northwest CAR.

Among the 206 surveyed female heads of household who reported an incident of sexual violence during their lifetime, 64% (95% CI 54.3 - 72.5) reported that the perpetrator was their husband or partner. For refugee female heads of households, 52.0% (95% CI 35.4 - 68.1) of sexual violence was perpetrated by a husband or partner compared to 71.1% (95% CI 61.0 - 79.5) for Cameroonian female heads of households.

The second most commonly identified perpetrators were soldiers/rebels or *coupeurs de route*. Of the 206 respondents who reported at least one incident of sexual violence during their lifetimes, 17.1% (95% CI 10.7 - 26.1) reported this type of perpetrator. Furthermore, this perpetrator was more commonly reported by refugee women, 39.0% (95% CI 25.6 - 54.2) compared to the host population 3.9% (95% CI 1.4 - 10.5). A total of eight female refugee heads of household reported multiple perpetrator sexual violence by soldiers/rebels and *coupeurs de route*.

Of the 206 female heads of household who reported sexual violence, 41 (20.0%, 95% CI 14.2 - 27.5) experienced sexual violence perpetrated by a friend or member of their community. Refugee survivors of sexual violence described the perpetrator as a friend or member of their community 15.6% (95% CI 8.3 - 27.2) of the time, while the affected female host population reported the perpetrator as friend or member of their community 21.2% (95% CI 13.9 - 32.8) of the time. Among refugees who reported sexual violence, 37.7% (95% CI 22.9 - 52.5) reported multiple perpetrators, while 6.3% (95% CI 1.6 - 10.9) of the host population who survived sexual violence reported multiple perpetrators.

A majority of both refugee and host population sexual violence survivors had not reported these events to the authorities. Only 24.7% (95% CI 13.8 - 40.1 of refugee women and 6.3% (95% CI 3.1 - 12.4) of host population sexual violence survivors had reported this sexual violence to any authority. Fourteen women surveyed, including eight refugees and six Cameroonians, reported ‘punishment’ for their perpetrators. These ‘punishments’ included being forced to marry the victim, payment, and in only three cases incarceration [17].

### Predictive value of human security indicators for gender-based violence

**Figure 1:**
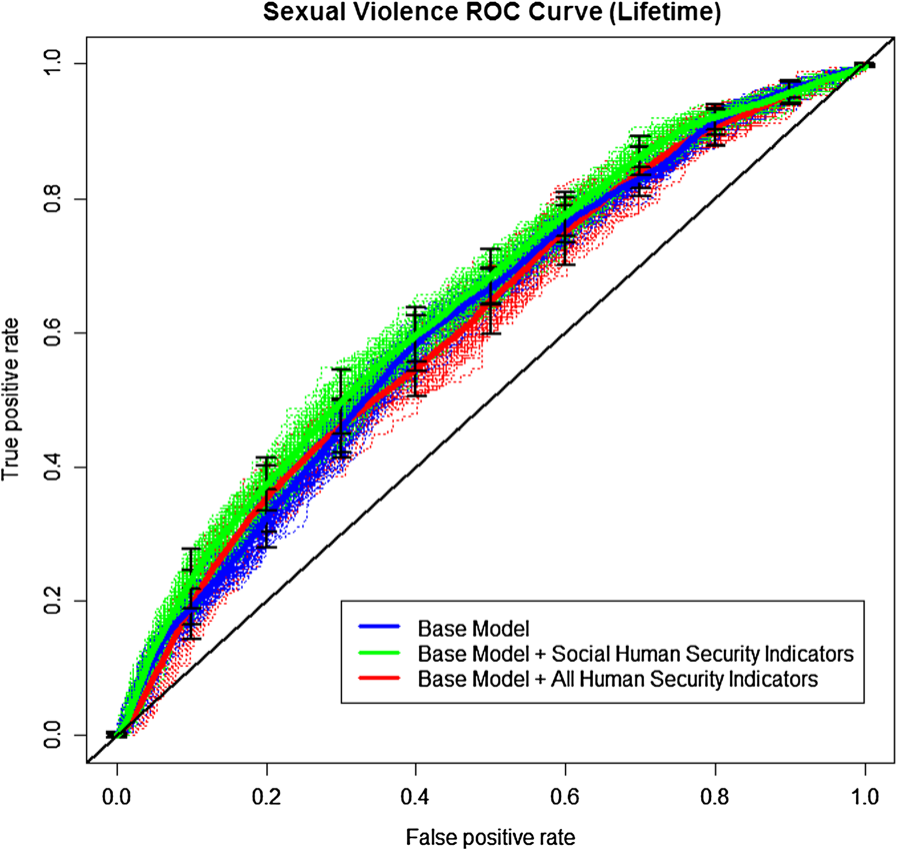
ROC for human security factors and lifetime sexual violence.

**Figure 2:**
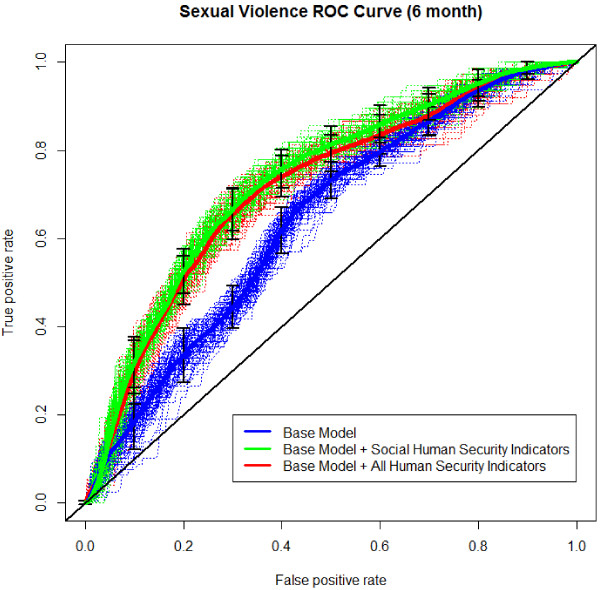
ROC for human security factors and six-month sexual violence.

The ability of human security indicators to predict the presence or absence of lifetime and six-month sexual violence was determined using receiver operating characteristic (ROC) analysis. The ROC curves, as shown in Figures 1 and 2, are generated using a five-fold cross-validation technique repeated 100 times. For each repetition, the folds are averaged to produce a single estimated ROC curve, and the 100 estimated ROC curves are averaged to produce an overall ROC curve. Figures 1 and 2 plot the ROC curves and the overall ROC curve (in bold) for lifetime and six-month prevalence of sexual violence, respectively, for three nested predictive models. The first model, titled ‘Base Model’, regresses sexual violence on predictors that include only age and ethnicity and achieves an area under the curve (AUC) of 0.62 and 0.64 for lifetime and six-month prevalence of sexual violence. The second model, titled ‘Base Model + Social Human Security Indicators’, contains the addition of sense of attachment to community human security indicators, and only achieves an AUC of 0.64 and 0.73 for lifetime and six-month prevalence of sexual violence respectively. The third model, titled ‘Base Model + All Human Security Indicators’, incorporates all human security factors (home, community, and future); however, these predictors did not improve prediction accuracy over the previous model (AUC of 0.62 and 0.71 for lifetime and six-month, respectively). Models 2 and 3 produced ‘poor’ and ‘fair’ predictions for the presence of lifetime or six month sexual violence, respectively, and only a modest gain in prediction over model 1, which used no human security indicators. These results provide evidence for the need to collect data that directly quantifies the level of sexual violence in a community.

## Discussion

Refugees and host population report similar levels of human security in terms of attachment to home, community, and a positive grasp of the future. Though some human security indicators differ between these populations, overall they reflect a relatively stable population at the time of this study, where refugees not only feel safe but also have access to land, livelihoods, clean water, shelter, and wish to remain in their newly adopted villages for many years to come. Refugees appeared to be able to attain stability and security in Eastern Cameroon—in fact, though the proportion of refugees that owned land was lower than the proportion of host population that owned land, refugees and Cameroonians who owned land owned in similar amounts, suggesting equal opportunity to grow livelihoods between the two groups. Similarly, refugees had equal levels of assets as many host population households. Refugees and Cameroonians were both similarly attached to their villages, desiring to stay in the same village well into the future.

However, despite the unusual and encouraging stability and egalitarian quality of these human security findings, these human security indicators missed an epidemic of sexual violence that endangered the lives and health of refugee and host population women. ROC analysis shows that human security indicators measured in this study did not uncover either lifetime or six-month sexual violence. These data suggest that current, gender-blind means of describing human security are missing serious threats to the safety of one half of the population. If human security is ‘concerned with the protection of people from critical and life-threatening dangers’ [25], this presents a major gap.

Why is it crucial that measures of human security be sensitive to gender? A robust body of evidence has explicitly linked measures of gender inequity and violence against women, including intimate partner and domestic violence, to higher levels of conflict [8,12,26-29]. Much of this work has been done by Caprioli, whose analyses have shown that increased domestic gender equity has a pacifying effect on state behavior at an international level [26], and that states with higher levels of gender equality are less likely to resort to violence first in resolving international disputes [27]. Caprioli went on in 2005 to link lower levels of gender equity to higher rates of internal conflict [8], and more violent conflict [28]. Melander reproduced these findings, demonstrating that societies with higher female representation in parliament and higher female to male education attainment ratios have lower levels of internal conflict [12]. There are several theories as to why this may be true. Some suggest that a tendency toward peaceful behavior is associated with a tolerance of the rights of others [29], while others cite that states with a tendency toward inequity and oppression are inherently likely to be more violent both internally and internationally [8,11]. Violence against women not only has implications for the human security of half of the world’s population, it has consequences for the stability of the state.

Human security indicators represent current feelings of respondents, and thus are not likely to be sensitive to distant events. Lifetime sexual violence could have occurred any time over the respondent’s lifetime, and in either CAR or Cameroon, making it difficult to interpret the meaning of high human security indicators in the context of a high prevalence of lifetime sexual violence. However, human security indicators were also not associated with any meaningful association with sexual violence during the past six months, suggesting an insensitivity of this model to a major threat to the security and health of women and girls. Further, lifetime and six month prevalence for sexual violence reported by both women in the CAR and Cameroon were similar, and both proportions were substantial. Sexual violence represents one extreme manifestation of gender inequity—this level of violence is likely to be associated with other manifestations, including limited access to education and access to justice. Low levels of literacy, low rates of completion of primary and secondary school education, poor access to justice for survivors of sexual violence, and high rates of early marriage were outlined in a previous publication focused on sexual violence findings of this study [17]. Thus, sexual violence against women represents a proxy indicator—it suggests that gender inequity has real human security consequences in this population, consequences that are not captured by this quantitative model, and consequences that extend beyond sexual violence itself.

These data also confirm what was described in the report of the Human Security Research Group published in 2012 [30]. This report outlined data suggesting that rape as a weapon of war, while important in many contexts, does not constitute the majority of rape faced by women in conflict. The findings of this report support this assertion [Tables 8 and 9], as the majority of women who experienced sexual violence reported that the perpetrator was their intimate partner (64.0%, 95% CI 54.3-72.5) and the second most common perpetrator was a friend or member of the community (20.0%, 95% CI 14.2-27.5). The group identified as ‘combatants’ - soldiers, rebels, and *coupers de route—*who, in most conflict contexts, would be the focus of sexual violence related insecurity—constituted a comparatively less commonly reported perpetrator (17.1%, 95% CI 10.7-26.1). When disaggregated data is examined [Tables 8 and 9], data show that while refugees report a higher proportion of sexual violence at the hands of combatants (39%, 95% CI 25.6-54.2) than Cameroonian women, refugees still report that a majority of the perpetrators of sexual violence are known to them (intimate partners: 52%, 95% CI 35.4-68.1: friends/community members: 15.6%, 95% CI 8.3-27.2). Based on these findings, we estimate 22.3% (95% CI 17.1-28.5) of all women in Djohong District, Cameroon have endured sexual violence by their intimate partner during their lifetimes. Several authors have reported high rates of sexual violence in times of conflict [31-34], and as suggested by the Human Security Research Group, much of that occurs at the hands of intimate partners and community members. While a focus on sexual violence perpetrated by armed forces remains important, the much more common epidemic of violence perpetrated by intimate partners and community members against women must also be addressed, particularly given that this violence presents risks not only for the affected women but for the development and maintenance of their communities and the world as a whole.

Several measures have been developed to understand gender equity at the country level, including UN Women’s Indicators and Statistics Database, GenderStats, and the World Economic Forum’s Gender Gap Project, and many studies analyze other measures including female to male education attainment ratio, female representation in parliament, life expectancy, literacy, and participation in democracy. However as Hudson points out, these do not include measures of physical violence against women [29]. These are also aggregated sources at the country level, and not likely to aid in a nuanced understanding of community level human security, as is the goal of the Leaning and Arie model.

Based on the findings of this study, we suggest that, at a minimum, measures of human security should include prevalence of sexual violence at the hands of all types of perpetrators, including both combatants and non-combatants. Additionally, measures of gender inequality that have been previously linked to conflict in other research (female participation in parliament and female to male higher education attainment ratio) should be included in human security frameworks.

### Limitations

All interviews were conducted privately in their homes in the respondent’s preferred language. Nuanced understanding of questions at the household level could be distorted in the course of translation and risked being asked differently in Fulfilde across data collectors. Though all efforts were made to ensure the respondents privacy at the time of the interview, it is possible that women felt reluctant to report sexual violence given strong social stigma associated with sexual violence. Whenever possible, respondents were interviewed by women, however, several members of the surveying team were male, due to limitations in availability of experienced multilingual staff. Although the few male data collectors had prior experience in medical and psychological intake of sexual violence survivors, it is possible that women felt less comfortable reporting their experiences with sexual violence to them regardless. We proceeded with male data collectors based on the acceptance in this community of male sexual violence counselors in a pre-existing international NGO program on sexual violence. Although female data collectors are recommended according to WHO guidelines [24], several studies have successfully engaged male data collectors in similar research [19,33]. As all data reported here refer to the respondent’s last episode of sexual violence, it is likely that these statistics under-represent the true burden of sexual violence in this community. Results are specific to refugee and host population female heads of household over the age of 18 only, thus results cannot be generalized to all women in the region and are likely biased towards the experiences of older women. Given these female heads of household tend to be first wives in polygamous households, this study represents the experiences of this group of women primarily. Arguably any form of sexual violence could affect human security and data was not collected on men or children.

Several of the data collectors were NGO sexual violence program staff and thus known to be affiliated with these programs. Although interviewers were carefully trained to emphasize that no aid or compensation would be given for participation in the survey, it is possible respondents may have altered their responses. Respondents may either have reported themselves to be refugees in the interests of obtaining services, or Cameroonians in order to avoid stigma. Given the anonymous nature of this survey and initial disclosure regarding aid and compensation, we do not feel it is likely that this issue significantly impacted responses.

Human security indicators were developed by the research team based on the Leaning-Arie model, used during a 2009 iteration of a population based survey in the region and refined based on the results. Outcomes are self-reported, thus assets, income, and land holdings were not verified objectively.

## Conclusion

This study suggests that in order to move toward an effective, accurate, and predictive quantitative measure of human security, measures of sexual and gender based violence must be included. A gender-inclusive model would not only ensure accurate measurement of what remains one of the main threats to women’s security, namely violence against women and girls both as a result of war and as a result of rape and physical violence in their homes and communities, but it would ensure a more sensitive means of understanding a community’s and state’s propensity toward violence. This will ultimately not only move us toward increasing the safety of women and girls, it could ultimately lead to a more secure and peaceful world.

## Competing interests

The authors have no competing interests to declare.

## Authors’ contributions

PKP was involved in study design, data acquisition and analysis of data, and primarily drafted the manuscript. PA was involved in field data acquisition, analysis of data, and manuscript preparation. JS was involved in analysis of data and manuscript preparation. RG directed statistical analysis of data and was involved in manuscript preparation. PGG was involved in study design, analysis of data, and manuscript preparation. All authors read and approved the final manuscript.
